# Supplementary material for: Phylogenomic Analysis of Two Co-Circulating Canine Distemper Virus Lineages in Colombia
Source: Pathogens. 2019 Dec 27;9(1):26. doi: 10.3390/pathogens9010026 (PMC7168600; doi:10.3390/pathogens9010026)
Supplement: Supplementary file 1 [file pathogens-09-00026-s001.pdf]

Supplementary Table 1. Substitutions associated with gastrointestinal symptoms

Protein	Position
N	449V, 465G
P	29V, 95R, 100N
C	88G
V	51L, 95R, 100N,143R
F	13V, 19L, 24R, 25S, 40K, 45F, 58Q, 64I, 72Y, 95P, 99G, 105W, 113C, 445P, 639R
H	38S, 241E, 291M, 325S, 333V, 365T, 487G, 530S

Supplementary Table 2. CDV Sites under positive selection

Protein site	dN	dS	Bayes factor	dN/dS
N-134	4.752	0.68	57.7	6.988
N-456	4.608	0.627	67.4	7.349
P-72	5.418	0.728	33.78	7.442
P-106	7.585	0.718	83.17	10.564
P-143	6.457	0.741	58.032	8.714
P-148	6.17	0.6859	67.527	8.995
P-195	6.013	0.681	58.412	8.830
P-221	6.123	0.668	63.472	9.166
P-237	4.641	0.588	42.048	7.893
P-287	4.196	0.587	47.292	7.148
P-296	4.047	0.68	24.756	5.951
V-72	4.465	0.626	24.267	7.133
V-90	2.82	0.587	11.454	4.804
V-106	5.924	0.634	45.958	9.344
V-143	4.106	0.683	21.431	6.012
V-148	5.739	0.578	59.668	9.929
V-195	5.741	0.613	55.469	9.365
V-221	3.924	0.622	20.431	6.309

V-235	3.493	0.619	18.332	5.643
V-237	2.835	0.576	11.792	4.922
C-3	9.158	0.538	822.524	17.022
C-37	3.789	0.661	11.962	5.732
C-45	5.164	0.625	30.715	8.262
C-86	4.998	0.661	16.887	7.561
C-91	9.03	0.654	123.251	13.807
C-99	5.901	1.052	17.952	5.609
C-169	7.423	0.65	57.385	11.420
C-171	4.37	0.667	19.927	6.552
M-9	4.921	0.591	32.626	8.327
M-294	5.606	0.64	59.512	8.759
F-21	7.141	0.781	60	9.143
F-53	7.199	0.695	87.613	10.358
F-72	6.631	0.709	62.9	9.353
F-98	6.919	1.365	26.563	5.069
F-99	6.608	0.796	50.091	8.302
F-101	6.828	0.817	42.661	8.357
F-102	6.105	0.714	49.482	8.550
H-549	10.547	1.037	272.214	10.171
